# Ketogenic Diet Decreases Emergency Room Visits and Hospitalizations Related to Epilepsy

**DOI:** 10.1155/2016/5873208

**Published:** 2016-09-26

**Authors:** Husam R. Kayyali, Anastasia Luniova, Ahmed Abdelmoity

**Affiliations:** ^1^Epilepsy Section, Neurology Division, Department of Pediatrics, Children's Mercy Hospitals and Clinics, University of Missouri in Kansas City, Kansas City, MO, USA; ^2^Neurology Department, Helen DeVos Children's Hospital, Grand Rapids, MI, USA

## Abstract

*Background*. Approximately, one-third of patients with epilepsy are refractory to pharmacological treatment which mandates extensive medical care and imposes significant economic burden on patients and their societies. This study intends to assess the impact of the treatment with ketogenic diet (KD) on reducing seizure-related emergency room visits and hospitalizations in children with refractory epilepsy.* Methods*. This is a retrospective review of children treated with the KD in one tertiary center. We compared a 12 months' period prior to KD with 12 months after the diet was started in regard to the number of emergency department (ED) visits, hospitalizations, and hospital days as well as their associated charges.* Results*. 37 patients (57% males) were included. Their ages at time of KD initiation were (4.0 ± 2.78) years. Twelve months after the KD initiation, the total number of ED visits was reduced by 36% with a significant decrease of associated charges (*p* = 0.038). The number of hospital admissions was reduced by 40% and the number of hospital days was reduced by 39%. The cumulative charges showed net cost savings after 9 months when compared to the prediet baseline.* Conclusion*. In children with refractory epilepsy, treatment with the ketogenic diet reduces the number of ED visits and hospitalizations and their corresponding costs.

## 1. Introduction

Epilepsy is a common neurological disorder affecting approximately 0.5–1% of the population in the United States. It is estimated that ~200,000 new cases per year are diagnosed in the United States [1]. Most patients with epilepsy achieve seizure control with antiepileptic medications. However, about one-third of patients suffer from refractory epilepsy [2]. This imposes a substantial economic burden on the health care system. In 2000, the burden of epilepsy was estimated to account for 0.5% of the total burden of diseases in the world [3]. Begley et al. estimated the annual cost for the 2.3 million prevalent epilepsy cases in the US in 1995 at $12.5 billion, with the direct costs being concentrated in patients with intractable epilepsy [4]. In 2016, this is about $19.5 billion when correcting the dollar value using CPI inflation calculator. Another study reported that annual direct medical costs of epilepsy per patient in the United States range from $1,620 to $52,558, depending on disease severity [5]. Drug-resistant epilepsy was found to be the most expensive category in a study from Italy, with the major sources of costs being hospital services followed by anticonvulsants [6].

The ketogenic diet (KD) is a well-established treatment modality for pharmacologically refractory epilepsy. Indeed, this diet is considered one of the oldest treatments for epilepsy in existence. In the 1920s, only phenobarbital and bromides were available for the treatment of epilepsy. As a result, the ketogenic diet was created in 1921 at the Mayo Clinic in Rochester, Minnesota, for children with refractory epilepsy [7]. Although initially it was very popular, as new anticonvulsants such as phenytoin were introduced, the popularity of the KD waned. In the 1990s, interest in the KD was reawakened again in the United States and then around the world [8]. Since then hundreds of publications have been devoted to the dietary treatment of epilepsy. The efficacy and safety of the KD in the treatment of epilepsy have been well established through many studies [9–12]. The ketogenic diet has been shown to be particularly effective in pediatric patients with refractory epilepsy [9, 13–16].

Despite its established efficacy, the data on the effect of the KD on health care costs and medical resource utilization is scarce. One case series compared the direct medical costs for 15 children with epilepsy before and after the diet. The authors reported a drop of the total costs for 6–12-month period from $352,820 before the diet to $190,659 (including $41,222 for diet initiation) [17].

We aim to study the effect of the KD treatment on the number of seizure-related emergency department (ED) visits, hospital admissions, hospital days, and their associated charges. This may shed some light on the KD potential to impact the health care costs of children with refractory epilepsy.

## 2. Methods

### 2.1. Study Design and Sample

This retrospective chart review included pediatric patients treated with the ketogenic diet for refractory epilepsy at a tertiary pediatric epilepsy center between 2009 and 2013. Institutional review board approval for the study protocol was obtained. The study cohort was selected based on the following inclusion criteria: (1) epilepsy was diagnosed and treated by a pediatric neurologist or epileptologist for ≥12 months prior to KD; (2) patients failed to respond to conventional pharmacological therapy before starting the diet; (3) patients were treated with the KD for ≥12 months.

### 2.2. Outcome Measures

We compared the 12-month period prior to starting ketogenic diet with the 12 months after the diet initiation in regard to the (1) number of ED visits; (2) number of hospital admissions; (3) number of hospital days; and (4) charges related to ED visits and hospitalizations.

### 2.3. Statistical Analysis

Descriptive statistics including means, medians, standard deviations, and proportions with 95% confidence interval were calculated. Paired *t*-tests were used to calculate *p* values. Wilcoxon Signed Rank tests were used to compare the 12 months before diet to the 12 months after diet for continuous variables such as total charges. The incidence rates of ED visits, inpatient visits, and hospital days were calculated by dividing the number of events by the patient months of observation. Incidence rate ratios (IRR) were then used to compare the prediet and postdiet periods where IRR < 1 indicates a lower incidence of the event in the postketogenic diet period.

## 3. Results

### 3.1. Patient Demographics

37 patients were included in this study (Table 1); 21 are males (57%). The average age at onset of epilepsy is 2.75 years (SD 1.46). The average age at KD initiation is 4 years (SD 2.78). The average duration of treatment with KD is 34.5 months (SD 17.89). The number of anticonvulsants used prior to initiating KD is 3–7 (mean 4.25), and on the last follow-up visit after the diet 0–4 (mean 2.32) AEDs were used.

### 3.2. Rates of ED Visits and Hospitalizations

The number of ED visits recorded for the entire cohort dropped from 66 visits in the 12 months before the diet to 42 visits in the 12 months after the diet. The incidence rate ratio (IRR) = 0.64 (95% CI 0.43–0.94). When ED visits related directly to seizures were analyzed separately, we found even more significant decline with IRR = 0.33 (95% CI 0.17–0.61) (Table 2). Individually, 26 patients (70.3%) experienced a decrease in their numbers of ED visits after the diet.

The same trend of decline was found in the number of hospitalizations; the total number of admissions decreased approximately by 40% (IRR = 0.60, 95% CI 0.39–0.92). The admissions related directly to epilepsy were decreased even more (IRR = 0.35, 95% CI 0.19–0.64). The number of hospital days also decreased at similar rates as summarized in Table 2. Out of the total cohort, 29 patients (78.4%) had a decrease in the number of their hospital admissions.

### 3.3. Charges Related to ED Visits and Hospitalizations

**Figure 1:**
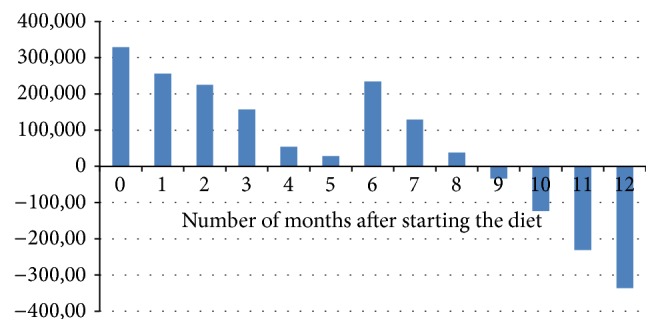
Cumulative net charges compared to prediet baseline. Net savings were achieved at 9 months after starting the diet and were maintained afterwards.

The average 12-month charges per patient are summarized in Table 3. The average ED charges decreased significantly in the post-KD period (*p* = 0.0386). Hospitalization charges were also significantly lower when excluding the charges related to the diet initiation (0.0052).  Figure 1 illustrates the cumulative cost difference from prediet baseline. The figure shows a trend of decreasing net charges until negative costs, which indicate net savings, were achieved 9 months after starting the diet.

## 4. Discussion

Despite recent advances in the management of epilepsy, poorly controlled seizures continue to be significant clinical problem affecting approximately 600,000 people in the United States [18]. Complications due to intractable epilepsy result in frequent hospitalizations and ED visits. The charges related to these services are “direct” costs that comprise a significant portion of the total health care costs imposed on refractory epilepsy patients. Although the clinical benefits of the ketogenic diet have been described extensively, information is lacking about its cost-effectiveness.

The diet was reported to have positive impact on the quality of life of pediatric patients which was attributed mainly to the decreased number of ED visits and decreased hospitalizations [19]. In this study we reemphasized the remarkable effect of the diet on lowering epilepsy-related ED visits and hospitalizations and subsequently on saving health care resources. The pre-post study design allowed us to eliminate intersubject variability by using patients as their own controls. Because of the unstable natural course of epilepsy, we elected to compare the immediate 12 months before diet initiation with the 12 months after initiating the diet for statistical purposes, knowing that the clinical effects of the diet appear usually after a variable period of several weeks after initiation. Even though we did not directly assess the clinical efficacy of the diet in the study cohort, it is reasonable to think that the significant reduction in the number of ED visits and hospitalizations is likely due to improved seizure control. This is supported by the results that show particularly remarkable decline in the hospitalizations and ED visits related to seizures compared to other reasons.

We found a steady decline of the charges related to hospitalizations and ED visits over the course of the 12 months after the diet initiation. The difference of the ED charges was statistically significant (*p* = 0.038). The ketogenic diet initiation requires 3–7-day admission to the hospital according to our epilepsy center protocol. We included the charges related to this hospitalization in the postdiet period. Despite the fact that our results showed a trend of decreasing net charges compared to the monthly average of the prediet period, negative costs, which indicate net savings, were achieved 9 months after starting the diet and were maintained afterwards.

Our analysis did not include other forms of health care costs like outpatient visit charges, cost of medications, laboratory and radiology tests, and so forth. For this reason, we acknowledge that our results do not necessarily prove the cost-effectiveness of the ketogenic diet. However, these results demonstrate the effectiveness of the diet in decreasing utilization of health care resources. An accurate assessment of the total cost of epilepsy requires studying both “direct costs” of medical resources devoted to diagnosing and treating patients and “indirect costs” from foregone earnings and reductions in household activities due to epilepsy-related morbidity and mortality [20]. The results of an ongoing randomized controlled trial evaluating the cost-effectiveness of a ketogenic diet are awaited [21]. Such studies are needed to shed further light on this subject.

## 5. Conclusion

This study demonstrates that, in children with refractory epilepsy, treatment with the ketogenic diet reduces the number of emergency department visits and hospitalizations due to seizure comorbidities, with a corresponding decline in the costs of these services.

## Figures and Tables

**Table 1:** Patients' characteristics.

Total number	37
Gender	
Male	21 (57%)
Female	16 (43%)
Age at epilepsy onset (yr)	2.75 ± 1.46^*∗*^
Age at KD initiation (yr)	4.0 ± 2.78^*∗*^
Duration of treatment with KD (mo)	34.67 ± 17.89^*∗*^
Number of AEDs used prior to KD	3–7 (mean 4.25)
Number of AEDs used after KD	0–4 (mean 2.32)

^*∗*^These values are the mean ± SD.

KD: ketogenic diet; AEDs: antiepileptic drugs.

**Table 2:** Rates of ED visits and hospitalization in the pre-KD period versus post-KD period.

	Before KD	Incidence rate (IR)^a^	After KD	Incidence rate (IR)^a^	IRR^b^	SD (95% CI)
*ED visits*	66	0.15	42	0.09	0.64	0.197 (0.43–0.94)
Seizure-related	40	0.09	13	0.03	0.33	0.319 (0.17–0.61)
Not seizure-related	26	0.06	29	0.07	1.12	0.27 (0.66–1.89)
*Hospitalizations*	55	0.12	33	0.07	0.60	0.22 (0.39–0.92)
Seizure-related	40	0.09	14	0.03	0.35	0.311 (0.19–0.64)
Not seizure-related	15	0.03	19	0.04	1.27	0.345 (0.64–2.49)
*Hospital days*	210	0.47	128	0.29	0.61	0.112 (0.49–0.76)
Seizure-related	152	0.34	55	0.12	0.36	0.157 (0.27–0.49)
Not seizure-related	58	0.13	73	0.16	1.26	0.176 (0.89–1.78)

^a^IR = incidence rate: it is the number of events divided by patient-months of observation.

^b^IRR = incidence rate ratio; IRR < 1 indicates a lower incidence of the event in the postketogenic diet period.

**Table 3:** 12-month charges per patient before and after ketogenic diet.

*n* = 37	Pre-KD period	Post-KD period	*p* value	Post-KD period excluding KD admission	*p* value
ED charges	$2,354 ± 5,935	$1,101 ± 3,586	0.0386	—	—
Inpatient charges	$33,675 ± 71,145	$30,889 ± 76,173	0.4014	$15,081 ± 70,828	0.0052
Total	$36,029 ± 76,179	$31,990 ± 78,488	0.4812	$16,183 ± 73,119	0.0036
